# Emerging Therapeutic Strategies for HPV-Related Cancers: From Gene Editing to Precision Oncology

**DOI:** 10.3390/cimb47090759

**Published:** 2025-09-15

**Authors:** Muharrem Okan Cakir, Guldide Kayhan, Betul Yilmaz, Mustafa Ozdogan, G. Hossein Ashrafi

**Affiliations:** 1 School of Life Sciences, Pharmacy and Chemistry, Kingston University London, London KT1 2EE, UK; m.okan@kingston.ac.uk; 2 School of Medicine, Medipol University, Istanbul 34810, Turkey; guldide.kayhan@std.medipol.edu.tr; 3 Department of Biochemistry, School of Medicine & Genetic and Metabolic Disease Research and Investigation Center, Marmara University, Istanbul 34722, Turkey; betulkarademir@marmara.edu.tr; 4 Department of Biochemistry, School of Medicine, Recep Tayyip Erdogan University, Rize 53100, Turkey; 5 Division of Medical Oncology, Memorial Hospital, Antalya 07050, Turkey; ozdoganmd@yahoo.com

**Keywords:** HPV-associated cancers, therapeutic vaccines, immune checkpoint inhibitors, CRISPR/Cas9, epigenetic drugs, natural compounds, drug repurposing, organoids, precision oncology

## Abstract

Human papillomavirus (HPV) is a major etiological factor in cervical, anal, and oropharyngeal cancers. Although prophylactic vaccines have substantially reduced infection rates, effective therapeutic options for established HPV-associated malignancies remain limited. This review provides an up-to-date overview of emerging strategies to treat HPV-driven tumours. Key approaches include immune checkpoint inhibitors, therapeutic vaccines such as VGX-3100 and PRGN-2012, and gene-editing tools like CRISPR/Cas9. Epigenetic drugs, particularly histone deacetylase inhibitors, show promise in reactivating silenced tumour suppressor genes and enhancing antitumour immunity. In addition, natural bioactive compounds and plant-derived molecules are being explored as complementary anti-HPV agents, while drug repurposing and combination therapies offer cost-effective opportunities to broaden treatment options. We also highlight the role of patient-derived organoid models as powerful platforms for personalized drug screening and functional assessment. By integrating these therapeutic innovations with precision oncology approaches, this review outlines a multidimensional framework aimed at improving clinical outcomes and quality of life for patients with HPV-associated cancers.

## 1. Introduction

**Figure 1:**
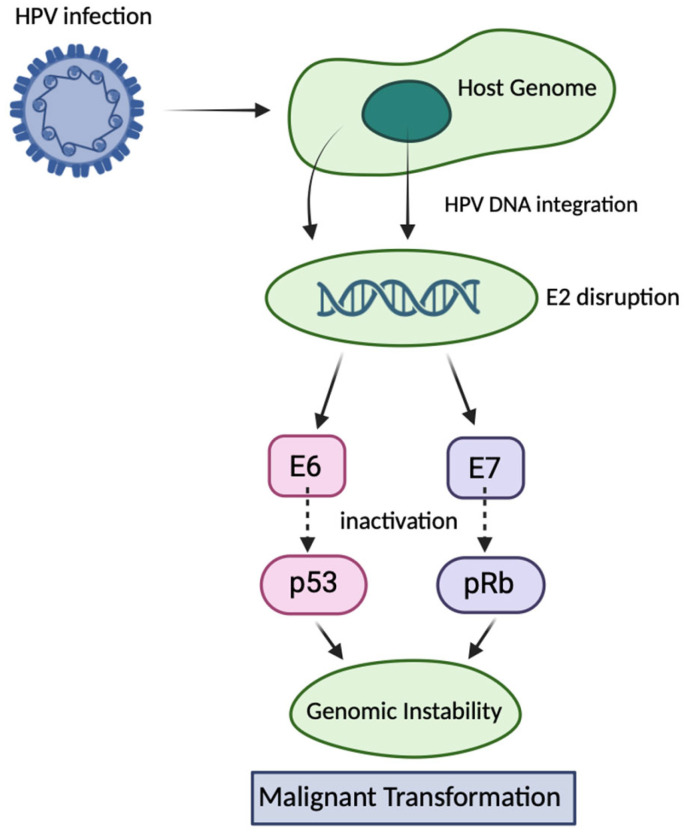
Mechanisms of HPV-induced carcinogenesis via E6 and E7 oncoproteins. Following infection of epithelial cells, high-risk human papillomavirus HPV integrates its DNA into the host genome, frequently disrupting the viral E2 gene. Loss of E2 function leads to uncontrolled expression of the viral oncoproteins E6 and E7. E6 promotes ubiquitin-mediated degradation of the tumour suppressor p53 via interaction with E6AP, whereas E7 binds and inactivates pRb, releasing E2F transcription factors. These events drive continuous cell cycle progression, impair DNA damage responses, and promote genomic instability, ultimately leading to malignant transformation.

Human papillomavirus (HPV) is a small, double-stranded DNA virus of approximately 8 kilobases, encapsulated by the structural proteins L1 and L2. More than 200 HPV types have been identified, of which around a dozen, including HPV-16 and HPV-18, are classified as high-risk due to their strong association with cervical, anogenital, and oropharyngeal cancers [1]. HPV primarily infects epithelial cells and can integrate its DNA into the host genome. This integration often disrupts the viral E2 gene, leading to uncontrolled expression of the viral oncoproteins E6 and E7. E6 forms a complex with E6-associated protein (E6AP) to mediate ubiquitin-dependent degradation of the tumour suppressor p53, while E7 inactivates retinoblastoma protein (pRb), releasing E2F transcription factors and driving uncontrolled cell proliferation. These molecular events promote genomic instability, a crucial step in malignant transformation [2] (Figure 1).

HPV also employs sophisticated immune-evasion strategies to establish persistence. Early viral proteins disrupt innate immune sensing and type I interferon pathways, while E6 and E7 downregulate major histocompatibility complex (MHC-I), reducing recognition by cytotoxic T lymphocytes [3,4]. Infected epithelia further remodel the tumour microenvironment by recruiting regulatory T cells and M2-polarized macrophages, fostering immunosuppression [5,6]. Although most HPV infections are transient and cleared within two years, approximately 10–30% persist, enabling long-term viral DNA retention, clonal expansion of infected cells, and progression to precancerous lesions or invasive cancers [7].

Prophylactic vaccines such as Gardasil and Cervarix induce neutralizing antibodies against the L1 capsid protein and are highly effective at preventing new infections. However, they lack therapeutic activity against established lesions [8]. Standard treatments, including surgery, radiotherapy, and chemotherapy, remove or destroy tumour tissue but do not eradicate integrated HPV DNA or reverse viral immune escape. Consequently, recurrence rates remain high, especially among patients with advanced disease or immunocompromised status [9,10]. Persistent or undetectable HPV DNA status after treatment has strong prognostic significance. Tumours lacking detectable HPV DNA show higher recurrence rates after radiotherapy, while a recent meta-analysis confirmed that HPV detectability is associated with improved survival outcomes in cervical cancer [11,12].

Given these limitations, novel therapeutic approaches are urgently needed. These include genome-editing platforms, epigenetic modulators, therapeutic vaccines, and natural bioactive compounds. In parallel, drug repurposing, organoid-based functional screening, and AI-enabled precision oncology are being developed to expand and personalize the therapeutic landscape. This review critically evaluates these emerging strategies and their translational potential for HPV-associated cancers.

## 2. Therapeutic Approaches

The development of effective treatments for HPV-associated cancers requires strategies that directly target viral oncogenes, reverse immune evasion, and personalize therapy to individual tumour profiles. Several therapeutic modalities are under investigation, including genome editing, epigenetic drugs, therapeutic vaccines, natural compounds, drug repurposing, and organoid-based functional screening. An overview of these approaches and their key characteristics is provided in Table 1.

### 2.1. Genome Editing Approaches

Gene-editing technologies such as Clustered Regularly Interspaced Short Palindromic Repeats/CRISPR-associated protein 9 (CRISPR/Cas9) and transcription activator-like effector nucleases (TALENs) directly target the viral oncogenes E6 and E7, which are essential for HPV-driven carcinogenesis. In vitro and in vivo studies have shown that disrupting these genes restores the function of the tumour suppressors p53 and pRb, leading to apoptosis, growth arrest, and tumour regression in mouse models. In vivo studies were primarily performed in HPV16+ tumour-bearing mouse models. Delivery methods included systemic intravenous or intraperitoneal administration, as well as subcutaneous injections. These approaches aimed to achieve tumour regression without targeting specific organs and are consistent across multiple preclinical reports [13,16].

**Figure 2:**
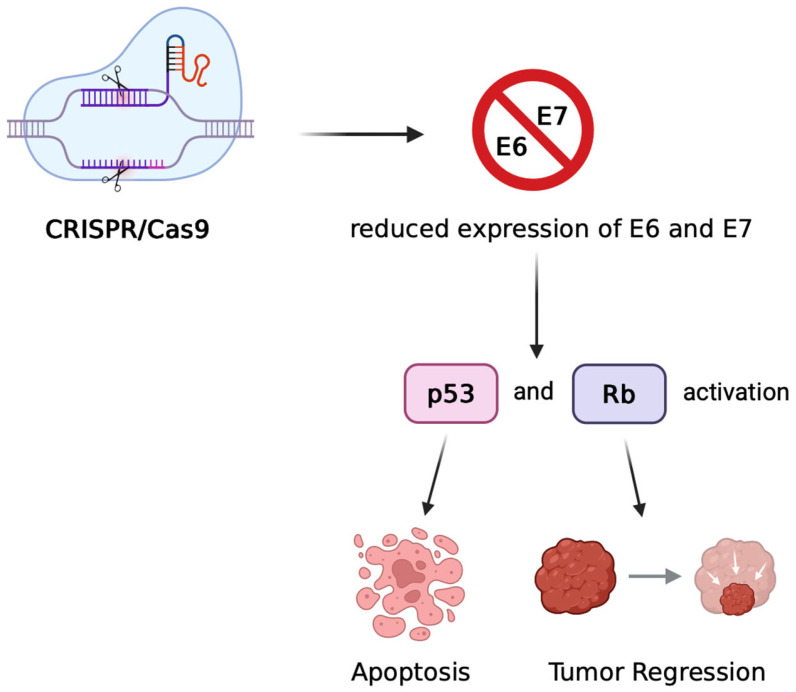
Mechanism of CRISPR/Cas9-mediated therapeutic targeting of HPV oncogenes. CRISPR/Cas9 introduces double-strand breaks in the HPV genome, leading to disruption of the E6 and E7 genes. The loss of E6/E7 expression restores the activity of tumour suppressor proteins p53 and pRb, resulting in cell cycle arrest, apoptosis, and tumour regression in preclinical models of HPV-associated cancers.

CRISPR/Cas9 is currently the most extensively studied platform for genome editing in HPV-associated cancers. Proof-of-concept experiments have demonstrated that CRISPR-mediated knockout of E6/E7 downregulates viral gene expression and suppresses tumour growth [13,16,17]. These effects are illustrated in Figure 2, and a comparison of two representative studies is summarized in Table 2.

Despite these promising findings, clinical translation remains limited by delivery and safety issues. Effective transport of genome-editing components is still the main barrier, with nanoparticles, liposomes, and viral vectors such as AAVs under active investigation [13,16,17,18]. Although high-fidelity Cas9 variants and optimized guide RNAs have reduced off-target effects, the risk of unintended edits persists [18,19]. Other genome-targeting strategies, including RNAi with siRNA or antisense oligonucleotides, can transiently suppress E6/E7 and partially restore p53 activity, but they lack the durable effects of CRISPR and have not yet progressed to human trials [20,21,22,23]. Overall, genome editing remains highly promising, but further work on safe delivery, off-target minimization, and regulatory considerations is required before translation into the clinic.

### 2.2. Epigenetic Drugs

High-risk HPV types, particularly HPV-16 and HPV-18, drive carcinogenesis not only through genetic disruption but also via extensive epigenetic reprogramming. The viral oncoproteins E6 and E7 increase DNA methylation and reduce histone acetylation, leading to silencing of tumour suppressor genes such as *p53* and *RB* and promoting immune evasion [24].

Epigenetic drugs have gained attention for their ability to reverse these oncogenic alterations. Histone deacetylase inhibitors (HDACi) such as vorinostat, romidepsin, and belinostat can reverse these changes, restore tumour suppressor expression and enhance tumour immunogenicity. In HPV-positive raft culture models, vorinostat suppressed E6/E7 activity, reduced viral genome amplification, and promoted apoptosis in differentiating keratinocytes [25]. Early-phase clinical studies are now exploring combinations of HDACi with immune checkpoint inhibitors or chemoradiotherapy in HPV-associated head and neck cancers [26].

**Figure 3:**
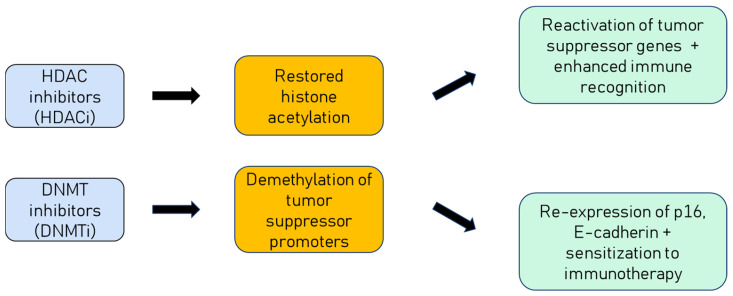
Mechanisms of epigenetic drugs in HPV-positive cancers. HDAC inhibitors restore histone acetylation, leading to reactivation of silenced tumour suppressor genes and enhanced immune recognition. DNMT inhibitors reverse hypermethylation, restoring expression of genes such as p16 and E-cadherin, and sensitizing tumours to immunotherapy.

DNA methyltransferase inhibitors (DNMTi) such as 5-azacytidine and decitabine can demethylate silenced promoters (e.g., p16, E-cadherin), restore antigen presentation, and sensitize tumours to PD-1/PD-L1 blockade. Preclinical work shows that DNMTi reduce E6/E7 expression and reactivate the p53/p21 axis [27]. Translational efforts now include a preoperative (window-of-opportunity) clinical trial evaluating 5-azacytidine, nivolumab, or their combination in resectable HPV-positive HNSCC (NCT05317000), aiming to enhance anti-tumour immunity via reversal of hypermethylation. These mechanisms are illustrated in Figure 3.

Epigenetic therapy provides a dual mechanism of action by reversing HPV-driven gene silencing and creating a tumour microenvironment that is more responsive to immune attack. These features position epigenetic drugs as promising components of multimodal regimens for HPV-associated malignancies.

### 2.3. Therapeutic Vaccines

Therapeutic vaccines aim to stimulate the immune system to eliminate HPV-infected or transformed cells by targeting the viral oncoproteins E6 and E7, which are consistently expressed in HPV-driven malignancies. Unlike prophylactic vaccines, such as Gardasil and Cervarix, that induce neutralizing antibodies against the L1 capsid protein, therapeutic vaccines are designed to elicit strong cytotoxic T-cell responses against established infections and lesions [28,29].

VGX-3100, a DNA-based vaccine encoding consensus sequences of HPV-16 and HPV-18 E6/E7 genes, has shown promising efficacy. Administered via intramuscular injection followed by electroporation, VGX-3100 induced robust antigen-specific CD8^+^ T-cell responses in women with cervical intraepithelial neoplasia grade 2 or 3 (CIN2/3). Phase I and II clinical trials demonstrated significant histologic regression and viral clearance, with durable immune responses lasting up to 24 weeks after vaccination [14,30]. The vaccine is currently undergoing phase III trials (REVEAL 1—NCT03185013 and REVEAL 2—NCT03721978) as well as combination studies with immune adjuvants and checkpoint inhibitors [14,31].

PRGN-2012, developed by Precigen and now known by its generic name Papzimeos (zopapogene imadenovec-drba)**,** is a gorilla adenovirus–based therapeutic vaccine designed to induce strong T-cell responses against HPV-6/11. It was initially tested in adult patients with recurrent respiratory papillomatosis (RRP), where a phase 1/2 trial (NCT04724980) demonstrated a ~51–55% complete response rate, with many patients avoiding surgical intervention for ≥12 months and showing improved vocal function, all with a favourable safety profile [32]. In August 2025, the U.S. Food and Drug Administration (FDA) granted full approval to Papzimeos for RRP which is the first immunotherapy approved for this rare HPV-driven disease, marking a landmark advance in the therapeutic vaccine field.

Despite encouraging results, several challenges remain, including optimization of antigen delivery methods, expansion of vaccine coverage beyond HPV-16/18 or 6/11, and overcoming immunosuppressive tumour microenvironments [18,33]. Combining therapeutic vaccines with checkpoint inhibitors, epigenetic drugs, or immune adjuvants is a promising strategy to enhance efficacy [28,29,33]. As ongoing clinical trials progress, therapeutic vaccines are poised to become an integral component of multimodal treatment regimens for HPV-driven diseases [28,33].

### 2.4. Natural Compounds & Phytochemicals

Plant-derived compounds continue to draw significant interest for modulating HPV oncogenesis and tumour immunity. The most extensively studied include curcumin, epigallocatechin gallate (EGCG), resveratrol, withaferin A, and berberine; more recent studies also highlight the soy isoflavone genistein and *Ficus carica* (fig) latex (Table 3). Mechanistically, these agents frequently downregulate E6/E7, restore p53/pRb signalling, trigger apoptosis and cell-cycle arrest, and may increase tumour immunogenicity—effects observed across in vitro and in vivo models [34,35,36,37,38,39,40,41,42,43].

Among the most extensively studied, curcumin, the active polyphenol in *Curcuma longa*, suppresses transcription of HPV16/18 E6 and E7, restores p53 and Rb activity, and inhibits malignant phenotypes in cervical cancer cells; nano-formulations improve its solubility and delivery, although clinical validation remains limited [40]. Epigallocatechin gallate (EGCG)**,** derived from green tea, has shown lesion regression in small clinical studies, but results from a larger randomized trial (Polyphenon-E) in women with persistent hrHPV/CIN1 did not demonstrate improved clearance, underscoring the need for optimized formulations and biomarker-guided patient selection [39,44]. Resveratrol, a polyphenol present in grapes and berries, has demonstrated inhibition of E6/E7 expression, induction of apoptosis, and suppression of cervical tumour growth in both in vitro and in vivo models [38]. Similarly, withaferin A, a steroidal lactone from *Withania somnifera*, downregulates E6/E7, restores p53, and induces apoptosis, with tumour growth reduction also observed in xenograft mouse models [37,45]. Another candidate, berberine, an isoquinoline alkaloid from *Berberis* species, represses E6/E7 expression, enhances p53/pRb activity, and triggers apoptosis via mitochondrial dysfunction and ROS accumulation [34,46]. More recently, the soy isoflavone genistein has been reported to reduce proliferation and modulate signalling pathways in cervical cancer cells, with emerging evidence confirming E6/E7 downregulation in HeLa cells [35,36]. A particularly noteworthy natural agent is fig latex (*Ficus carica*)**,** which our group and others have shown to exert selective cytotoxicity against HPV-positive cervical cancer cells. Fig latex downregulates E6/E7, reactivates p53/Rb, induces cell-cycle arrest, and reduces migration and invasion, while sparing normal keratinocytes. Transcriptomic profiling further demonstrated enrichment of antigen-presentation pathways and regulation of genes involved in cell-cycle control, supporting its potential to enhance tumour immunogenicity [41,42,43,47].

Despite robust in vitro and in vivo evidence, translation into clinical practice remains challenging. Bioavailability, extract standardization, and variability in pharmacokinetics limit consistent efficacy, and the absence of large-scale controlled trials hampers definitive conclusions. Future efforts must therefore focus on pharmacological optimization, rigorous preclinical validation, and well-designed clinical studies to fully establish the therapeutic potential of natural compounds in HPV-associated malignancies.

## 3. Drug Repurposing & Combination Therapies

The development of entirely new drugs is often time-consuming and costly. Drug repurposing, which involves identifying new therapeutic uses for existing approved drugs, has therefore emerged as a promising strategy for the treatment of HPV-related cancers. Its key advantages include lower cost, shorter development timelines, and well-established safety profiles. Conceptual frameworks and curated databases (e.g., ReDO/ReDO_DB) continue to map non-oncology agents with anticancer potential [48,49].

Several repurposed drugs have shown promise in HPV-driven malignancies. Niclosamide, an antihelminthic, inhibits E6/E7 expression, disrupts Wnt/β-catenin and mTOR signalling, induces mitochondrial stress, and synergizes with paclitaxel in cervical cancer cells [50]. Lopinavir, an HIV protease inhibitor, demonstrated cytotoxicity in HPV-transformed cells and, in a proof-of-concept study, topical self-applied lopinavir gel induced regression of CIN2/3 lesions in a subset of women [51]. Cidofovir, an antiviral DNA polymerase inhibitor, has been applied topically or intralesionally in HPV-associated neoplasia and recurrent respiratory papillomatosis, with clinical responses reported [52,53]. In addition, GS-9191, a topical nucleotide analogue (prodrug of PMEG), potently inhibited HPV-positive cells in vitro and reduced papilloma burden in preclinical models, with early clinical testing in anogenital warts (NCT00499967) [54].

In parallel, combination strategies are being explored to overcome resistance and enhance efficacy. These include chemo-immunotherapy, epigenetic drugs combined with immune checkpoint inhibitors, and therapeutic vaccines administered alongside immunomodulatory agents. A notable example is a window-of-opportunity phase II trial (NCT05317000) testing 5-azacytidine, nivolumab, or their combination in resectable HPV-positive head and neck cancers, aiming to reverse promoter hypermethylation and augment antitumour immunity. Moreover, PD-1 inhibitors such as nivolumab are already approved for recurrent/metastatic HNSCC after platinum therapy (CheckMate-141), highlighting the clinical relevance of immunotherapy in HPV-driven disease.

An overview of representative investigational and approved therapies is provided in Table 4, while Table 5 summarizes their key mechanisms of action.

Moving forward, successful translation will require biomarker-driven patient stratification, larger randomized studies, and careful integration of repurposed agents into multimodal frameworks. If validated, these strategies could accelerate the delivery of effective and personalized therapies for patients with HPV-associated malignancies.

## 4. Patient-Derived Organoids & Functional Screening

Organoids are three-dimensional (3D) miniaturized tissue cultures derived either from pluripotent stem cells or directly from patient tumour biopsies. They closely recapitulate the genetic, phenotypic, and architectural complexity of the original tumour, offering substantial advantages over conventional two-dimensional (2D) monolayers [55]. Stem cell–derived organoids are particularly useful for modelling epithelial differentiation and viral infection, while patient-derived tumour organoids retain the mutational and phenotypic landscape of HPV-driven cancers, including cervical, oropharyngeal, and anal malignancies [56,57].

HPV-positive tumour organoids faithfully retain hallmark oncogenic drivers such as E6/E7 expression, genomic instability, and immune evasion signatures, thereby serving as physiologically relevant ex vivo models to study viral persistence, host–virus interactions, and HPV-related tumour biology [57]. Importantly, they also enable testing of therapeutic responses in a patient-specific context, offering a functional precision medicine tool for HPV-associated cancers [56].

Drug screening on organoids allows high-throughput evaluation of chemotherapeutics, targeted agents, immunotherapies, and natural compounds under conditions that better mimic the tumour microenvironment compared to 2D cultures. Coupled with pharmacogenomics, these assays can map drug sensitivities across HPV genotypes and patient subgroups, aiding biomarker discovery and therapy stratification [55,57]. Furthermore, integrating organoid-derived datasets with artificial intelligence (AI) and machine learning accelerates predictive modelling, linking multi-omic and transcriptomic profiles to drug responses. Recent examples include PharmaFormer, a transfer-learning framework that uses organoid pharmacogenomic data to predict clinical drug responses, and multi-omic ML models achieving high accuracy in predicting therapy outcomes [58,59]. These approaches highlight the potential of AI-enhanced organoid platforms to transform personalized oncology in HPV-driven cancers.

A frequently raised limitation is the time required for organoid establishment, which may take several weeks. While this is valid, organoid biobanks and automation are increasingly mitigating this concern. Large-scale repositories have been established for colorectal, gastric, and breast cancers, capturing both inter- and intra-patient heterogeneity and enabling rapid drug testing [60,61]. Similar efforts are underway for HPV-related malignancies, with oral and cervical mucosal organoid panels providing proof-of-concept for HPV-associated cancers [62]. These pre-established biobanks allow near-immediate access to patient-matched models, bypassing lengthy culture times. In parallel, automation and microfluidic systems enable parallel organoid generation and high-throughput drug screening, ensuring clinically actionable results within relevant timelines [63]. Together, these innovations directly address feasibility concerns and make organoid-based precision oncology increasingly compatible with real-world patient care.

Overall, patient-derived organoid models represent a transformative tool for HPV-related cancer research. By faithfully reproducing patient-specific tumour biology, they bridge fundamental virology with translational precision medicine, enabling functional drug testing, biomarker discovery, and the design of individualized therapeutic regimens [56,57,60].

## 5. Precision Oncology in HPV Associated Cancers

Precision oncology in HPV-related cancers increasingly leverages viral, host, and immune biomarkers to guide therapy. PD-L1 expression is predictive of immune checkpoint inhibitor (ICI) response in recurrent/metastatic head and neck cancers [64]. APOBEC mutational signatures are enriched in HPV-positive HNSCC and have been linked to prognosis and immunotherapy sensitivity [65].

HPV DNA integration correlates with oncogene activation and poor prognosis [66,67]. p16^INK4a^ overexpression is a robust surrogate of oncogenic HPV activity and remains a standard clinical marker [68]. In addition, circulating HPV DNA (cfDNA) has emerged as a promising biomarker for minimal residual disease monitoring and relapse detection in cervical and oropharyngeal cancers [69].

A summary of key biomarkers used in HPV precision oncology is provided in Table 6, highlighting their clinical applications and translational potential.

## 6. Artificial Intelligence in HPV-Associated Cancers

Artificial intelligence (AI) is increasingly being applied to HPV-associated cancers to improve biomarker integration and therapy prediction (Table 7). Deep learning applied to histopathology can accurately predict HPV status and survival outcomes in oropharyngeal cancers [70]. Machine learning models integrating transcriptomic and immune profiles (e.g., random forest classifiers) have been shown to distinguish between high-grade lesions and invasive cervical cancer with strong accuracy [71]. Single-cell immune profiling indicates that CD161^+^ tissue-resident memory T-cells may serve as predictors of immunotherapy response in HPV-positive HNSCC [72]. In addition, AI analysis of circulating HPV DNA kinetics can detect recurrence earlier than conventional imaging, offering a valuable tool for MRD monitoring [73].

## 7. Conclusions and Future Directions

Therapeutic strategies for HPV-related cancers have diversified markedly in recent years, extending from genome editing to immunotherapy, therapeutic vaccines, drug repurposing, natural compounds, and patient-derived models. Despite encouraging preclinical results, translation into routine clinical practice remains limited. Major barriers include inefficient and potentially unsafe delivery of genome editor, the immunosuppressive tumour microenvironment that restricts vaccine and checkpoint inhibitor efficacy, poor bioavailability of phytochemicals, and the absence of validated biomarkers for therapy stratification [15,74,75,76,77].

Several clinical trials illustrate the movement of these approaches into translational settings. Table 8 summarizes selected examples, including immune checkpoint inhibitors, therapeutic vaccines, and early gene-editing interventions. These efforts mark important steps toward clinical validation, even though most remain in phase I–II.

Despite these advances, limitations persist. Natural compounds demonstrate antiviral and antitumour effects in vitro but lack consistent pharmacokinetic profiles and large-scale clinical validation [15]. Repurposed drugs such as niclosamide show synergy with chemotherapy in preclinical models but remain under early investigation [50]. Organoid models offer patient-specific drug screening potential yet require greater standardization for clinical implementation. Cervical cancer organoids recapitulate HPV integration and heterogeneity, lesion-derived organoids enable co-culture immunotherapy testing, and organoid biobanks are emerging as powerful translational resources to evaluate therapy response across patient subgroups [78,79,80].

Meanwhile, AI applications in HPV-related cancers are emerging: deep learning can predict HPV status from pathology slides and machine-learning models integrating transcriptomic and immunogenomic data are beginning to predict immunotherapy response [70,71,72,81].

Future progress will depend on integration rather than isolated strategies. Combining genome editing with immunotherapies or therapeutic vaccines may overcome resistance and heterogeneity. Organoid biobanking and automated high-throughput drug screening can shorten timelines for patient-specific testing, while AI-driven biomarker discovery has the potential to unify molecular, transcriptomic, and clinical data. Clinical trial frameworks must also evolve by adopting adaptive, basket, and umbrella designs to evaluate multimodal interventions in HPV-driven malignancies [82].

Aligning molecular biology, immunology, bioinformatics, and clinical oncology may transform HPV-associated cancers from treatment-refractory conditions into exemplars of precision-guided, integrative care in the coming decade.

## Figures and Tables

**Table 1:** Overview of Therapeutic Strategies Targeting HPV-Driven Cancers [13,14,15].

Approach	Mechanism	Advantages	Challenges
**CRISPR/TALEN**	E6/E7 gene disruption → p53/pRb reactivation	Durable effect, tumour suppression	Vector delivery, off-target effects, safety concerns
**RNAi**	Suppression of E6/E7 mRNA	Transient, no genomic alteration	Short-lived effects, delivery difficulties
**Therapeutic Vaccines**	Induction of T cell immune response	Immune activation, long-term protection	Limited Phase III data
**Small Molecules**	Inhibition of oncoprotein complexes	Targeted, direct molecular intervention	Potential off-target effects on normal cellular functions
**Nano-therapy**	Targeted drug delivery	Increased efficacy, reduced toxicity	Complex manufacturing, safety uncertainties
**Organoids + AI**	Personalized drug response modelling	Precision medicine, biomarker discovery	Lack of standardization, limited datasets

**Table 2:** Comparison of Representative CRISPR-Based Approaches Targeting HPV Oncogenes.

**Feature**	[16]	[13]
Model	In vitro (SiHa, CaSki cell lines)	In vivo (HPV16+ tumour-bearing mice)
Target	E7 oncogene only	Primarily E7, but also systems targeting both E6 and E7
Delivery Method	Plasmid-based transfection	Systemic delivery via PEGylated liposomes
Outcome	Apoptosis induction, pRb restoration, growth arrest	Tumour regression, prolonged survival, efficient genome editing
Challenges	Limited to in vitro application	Need for metastatic targeting, off-target risks, vector optimization

Vector optimization is required to enhance delivery efficiency, ensure tumour-specific targeting, minimize off-target effects, and improve safety for clinical translation.

**Table 3:** Selected natural compounds with anti-HPV activity and mechanisms of action.

Compound	Natural Source	Mechanism of Action	HPV-Related Effect	References
Curcumin	*Curcuma longa*	↓ E6/E7 transcription; restores p53/Rb; anti-proliferative	Suppresses HPV oncogenes; growth/migration ↓ (in vitro/in vivo)	[40]
EGCG	Green tea	Antioxidant; epigenetic effects; cell-cycle arrest	Lesion responses in small studies; mixed RCT results	[39,44]
Resveratrol	Grapes/berries	↓ E6/E7 transcription; anti-proliferative	Inhibits cervical cancer growth (in vitro/in vivo)	[38]
Withaferin A	*Withania somnifera*	↓ E6/E7; p53 restoration; G2/M arrest; apoptosis	Tumour growth ↓ in mice; pro-apoptotic signalling	[37,45]
Berberine	*Berberis* spp.	Mitochondrial dysfunction; ↑ ROS; apoptosis; ↓ E6/E7	Triggers apoptosis in HPV-transformed cells	[34,46]
Genistein	Soy isoflavone	Antiproliferative; pathway modulation; (new) ↓ E6/E7	Reduces proliferation; emerging E6/E7 data	[35,36]
Fig latex	*Ficus carica*	↓ E6/E7; re-activates p53/Rb; cell-cycle arrest; ↑ antigen presentation	Selective cytotoxicity vs. HPV+ cells; migration/invasion ↓; immunogenicity ↑	[41,42,43,47]

“↑” denotes upregulated expression (relative to control). “↓” denotes downregulated expression (relative to control).

**Table 4:** Investigational and Approved Therapies Targeting HPV Pathways [14,50,51,52,53,54].

Drug/Therapy	Type	Target/Mechanism	Clinical Phase
Nivolumab	Immune Checkpoint Inhibitor	PD-1	Approved for recurrent/metastatic HNSCC (FDA 2016)
Decitabine	Epigenetic Drug	DNMT inhibition	Phase II window trial in HPV + HNSCC (NCT05317000)
VGX-3100	DNA Therapeutic Vaccine	E6/E7-specific immune response	Phase III (REVEAL program)
CRISPR-E7 (preclin)	Gene Editing	E7 knockout via Cas9	Preclinical
Curcumin	Natural Compound	NF-κB inhibition, apoptosis induction	Preclinical

**Table 5:** Mechanisms of action of selected anti-HPV therapeutics [32,50,51,52,53,54].

Drug	Mechanism of Action
VGX-3100	DNA vaccine inducing E6/E7-specific T-cell responses; phase IIb efficacy in CIN2/3; phase III ongoing
Lopinavir/Ritonavir	HIV protease inhibitor: topical application showed regression/HPV clearance signal in CIN2/3
Niclosamide	Downregulates E6/E7; induces autophagy, ROS-mediated mitochondrial stress; inhibits Wnt/β-catenin and mTOR signalling
Cidofovir	Viral DNA polymerase inhibitor; topical/intralesional use for HPV-related lesions and papillomatosis
PRGN-2012	Gorilla adenovirus vaccine; induces HPV-6/11-specific T cells; durable responses in RRP; FDA-approved in 2025 as first immunotherapy for RRP
GS-9191	Topical nucleotide analogue (PMEG prodrug); inhibits viral DNA synthesis; early clinical testing (NCT00499967)

**Table 6:** Key biomarkers in precision oncology for HPV-associated cancers.

Biomarker	Cancer Type	Clinical Application	Reference
PD-L1 expression	Cervical, HNSCC	Predictive for ICI response	[64]
Tumour mutational burden (TMB)	Cervical, HNSCC	Immunotherapy stratification	[65]
APOBEC signature	HPV + HNSCC	Prognosis, immunotherapy sensitivity	[65]
HPV DNA integration	Cervical, HNSCC	Prognosis, oncogene activation	[66,67]
p16^INK4a^	Cervical, HNSCC	Surrogate marker for oncogenic HPV	[68]
Circulating HPV DNA	Cervical, HNSCC	MRD monitoring, relapse detection	[69]

**Table 7:** AI applications in HPV-associated cancers.

AI Approach	Input Data	Application	Reference
Deep learning on histology	H&E slides (HNSCC)	HPV status classification, survival strat.	[70]
ML (Random Forest)	Transcriptome + immune data	Lesion progression, ICI response prediction	[71]
Single-cell immune profiling	HPV + HNSCC tumours	Predict immunotherapy response	[72]
AI on cfDNA kinetics	Plasma HPV DNA	MRD monitoring, early recurrence detection	[73]

**Table 8:** Selected clinical trials in HPV-related cancers.

Trial/NCT	Intervention	Indication	Status/Key Notes
CheckMate-358 (NCT02488759)	Nivolumab (PD-1)	R/M cervical, vulvar	Completed; ORR ~26% in cervical cohort [76]
REVEAL-1 (NCT03185013)	VGX-3100 (E6/E7 DNA vaccine) + EP	Cervical HSIL (CIN2/3)	Completed phase 3; built on effective phase 2b [14]
REVEAL-2 (NCT03721978)	VGX-3100 (E6/E7) + EP	Cervical HSIL	Completed phase 3; pending publication outcomes
NCT03057912	TALEN/CRISPR-Cas9 targeting E6/E7	Persistent HPV, CIN I	Withdrawn pre-enrolment—underscores delivery/safety limitations
NCT05334004	Lopinavir/ritonavir (intra-anal)	HGAIN/AIN 2–3	Ongoing phase I safety trial

## Abbreviations

The following abbreviations are used in this manuscript:

AI	Artificial Intelligence
AAV	Adeno-Associated Virus
CIN	Cervical Intraepithelial Neoplasia
CRISPR	Clustered Regularly Interspaced Short Palindromic Repeats
Cas9	CRISPR-associated protein 9
cfDNA	Circulating Cell-free DNA
DNMTi	DNA Methyltransferase Inhibitor
EGCG	Epigallocatechin Gallate
FDA	U.S. Food and Drug Administration
HDACi	Histone Deacetylase Inhibitor
HPV	Human Papillomavirus
HNSCC	Head and Neck Squamous Cell Carcinoma
ML	Machine Learning
mTOR	Mechanistic Target of Rapamycin
NMD	Nonsense-Mediated Decay
PDO	Patient-Derived Organoid
pRb	Retinoblastoma Protein
RRP	Recurrent Respiratory Papillomatosis
ROS	Reactive Oxygen Species
siRNA	Small Interfering RNA
TALEN	Transcription Activator-Like Effector Nuclease
TME	Tumour Microenvironment
VEGF	Vascular Endothelial Growth Factor
